# Dataset for transcriptomic, H3K9ac and H3K9me3 profiles during cardiac regeneration

**DOI:** 10.1016/j.dib.2022.108569

**Published:** 2022-09-02

**Authors:** Xuelong Wang, Huiping Guo, Feifei Yu, Hui Zhang, Ying Peng, Chenghui Wang, Gang Wei, Jizhou Yan

**Affiliations:** a Department of General Surgery, Pancreatic Disease Center, Ruijin Hospital, Shanghai Jiaotong University School of Medicine, Shanghai, China; b Department of Developmental Biology, and Institute for Marine Biosystem and Neurosciences, Shanghai Ocean University, Shanghai, China; c CAS Key Laboratory of Computational Biology, Chinese Academy of Sciences, Shanghai, China; d Department of Aquaculture, Shanghai Ocean University, Shanghai, China; e Present address: School of Life Sciences and Technology, Tongji University, Shanghai, China.

**Keywords:** H3K9ac, H3K9me3, RNA-seq, ChIP-seq, Epigenetics, Blastema

## Abstract

Acetylation and tri-methylation of histone H3 lysine 9 (H3K9ac and H3K9me3) play an interactive regulatory role in the epigenetic regulation of gene expression during heart development and cardiovascular disease, but little is known about their possible role in heart regeneration. Here we utilized genome-wide high-throughput RNA sequencing (RNA-seq) and chromatin immunoprecipitation with high-throughput sequencing (ChIP-seq) for H3K9ac and H3K9me3, carried out on regenerative cardiac tissues at different days post amputation in zebrafish (*Danio rerio*) to investigate dynamic changes in gene expression and the epigenetic landscape of H3K9ac and H3K9me3. The STAR, Bowtie2, MACS2, and deepTools2 were mainly used for RNA-Seq or ChIP-seq data analysis. In this article, we present detailed information on experiment design, data generation, quality assessment and processing pipeline. Raw reads of the RNA-seq and ChIP-seq data have been deposited at the NCBI GEO repository with the accession number GSE158104. Our data will be a valuable resource for the elucidation of H3K9ac and H3K9me3 involvement in the regulation of gene transcription during cardiac regeneration.

**Specifications Table**

**Table:** 

Subject	Molecular Biology
Specific subject area	Epigenetics
Type of data	Tables
How data were acquired	Library construction of RNA-seq, H3K9ac and H3K9me3-based chromatin immunoprecipitation (ChIP), followed by sequencing and bioinformatics.
Data format	Raw
Description of data collection	The RNA and ChIP-DNA were isolated from regenerative cardiac tissue at 0, 6, 9 days post amputation (dpa) in zebrafish. Sequencing of RNA-seq and ChIP-seq libraries was performed on the Illumina HiSeq 2500 platform with 2 × 150 bp paired-end reads. On average, 25-30 million raw paired reads were obtained for each sample of RNA-seq library, and on average, 30-50 million paired-end reads were obtained for each sample of ChIP-seq library using Illumina stranded sequencing.
Data source location	Department of Developmental Biology, Institute for Marine Biosystem and Neurosciences, Shanghai Ocean University, China
Data accessibility	Raw and processed data of RNA-seq and ChIP-seq has been deposited at the NCBI GEO repository under the accession number GSE158104 (https://www.ncbi.nlm.nih.gov/geo/query/acc.cgi?acc=GSE158104).
Software and programs	FastQC (http://www.bioinformatics.babraham.ac.uk/projects/fastqc/), STAR [1], Cufflink [2], SAMtools [3], Bowtie2 [4], MACS2 [5], deepTools2 [6].
Related research article	Wang, X., Guo, H., Yu, F., Zhang, H., Peng, Y., Wang, C., Wei, G., & Yan, J. Keratin5-cytoskeleton-BMP4 network regulates cell phenotype conversions during cardiac regeneration. Experimental Cell Research, 2022, 418(1), 113272. DOI: https://doi.org/10.1016/j.yexcr.2022.113272

## Value of the Data

• Our data represents valuable and unique insight into histone modifications, including H3K9ac and H3K9me3, as well as differential gene expression during cardiac regeneration at 0 dpa, 6 dpa and 9 dpa in zebrafish.
• The reported dataset is the combined data on ChIP-seq for H3K9ac and H3K9me3 and RNA-seq, which allows tracing the relations between characteristics of H3K9as and H3K9me3 signals, as well as mRNA expression at the level of individual genes, providing a valuable resource for regeneration researchers.
• The sequencing data could be used to examine the impact of acetylation and tri-methylation of histone H3 lysine 9 in individual nucleosomes on the expression efficiency of the corresponding gene during cardiac regeneration, and facilitates the molecular understanding of heart regeneration-related genes.

## 1 Data Description

This article reports the sequence data of mRNA and ChIP for H3K9ac and H3K9me3 from the regenerative cardiac tissues at 0, 6 and 9 days post amputation in zebrafish. Two biological replicates were done for each RNA sample, and ChIP-seq experiments were conducted without biological replicates. The raw data (fastq files) has been deposited at the NCBI GEO repository under the accession number GSE158104 (https://www.ncbi.nlm.nih.gov/geo/query/acc.cgi?acc=GSE158104). The quality of raw sequencing reads, including basic statistics, per base sequence quality and content, adapter content, was evaluated using FastQC. The HTML output files of FastQC are provided as supplementary materials. Detailed information on mRNA sequencing data is summarized in Table 1 and the ChIP sequencing data for H3K9ac and H3K9me3 are summarized in Tables 2 and 3, respectively.

**Table 1:** Statistics: RNA-seq data of cardiac regeneration in zebrafish

RNA-seq samples	0dpa_rep1	0dpa_rep2	6dpa_rep1	6dpa_rep2	9dpa_rep1	9dpa_rep2
Number of input reads	26000581	25509314	26074373	28564974	25861288	27410762
Uniquely mapped reads number	22787288	23142429	23295067	25130009	23282152	24657314
Uniquely mapped reads %	87.64%	90.72%	89.34%	87.97%	90.03%	89.95%
Mismatch rate per base, %	0.58%	0.60%	0.60%	0.63%	0.64%	0.60%
Number of reads mapped to multiple loci	1813298	935344	1277636	1805766	1095297	1111390
% of reads mapped to multiple loci	6.97%	3.67%	4.90%	6.32%	4.24%	4.05%

**Table 2:** Statistics: ChIP-seq data for H3K9ac of cardiac regeneration in zebrafish

ChIP-seq samples	H3K9ac_0dpa	H3K9ac_6dpa	H3K9ac_9dpa
Number of input reads	38027847	30388283	40542596
aligned concordantly exactly 1 time	25003706 (65.75%)	19840450 (65.29%)	26102930 (64.38%)
aligned concordantly >1 times	7722612 (20.31%)	6377034 (20.99%)	8150424 (20.10%)
overall alignment rate	90.81%	90.91%	89.46%

**Table 3:** Statistics: ChIP-seq data for H3K9me3 of cardiac regeneration in zebrafish

ChIP-seq samples	H3K9me3_0dpa	H3K9me3_6dpa	H3K9me3_9dpa
Number of input reads	34407755	50182174	40891425
aligned concordantly exactly 1 time	10417534 (30.28%)	24916143 (49.65%)	22109776 (54.07%)
aligned concordantly >1 times	5114316 (14.86%)	10671763 (21.27%)	9309377 (22.77%)
overall alignment rate	50.12%	75.62%	82.68%

## 2 Experimental Design, Materials and Methods

### 2.1 RNA isolation, library preparation and sequencing

Total RNAs were isolated from the newly regenerated heart tissues of zebrafish at 0 dpa, 6 dpa and 9 dpa using TRIzol Reagent (Invitrogen, USA) and further purified using RNeasy Mini Kit (Qiagen, Germany) and ran on Agilent Bioanalyzer 2100 to assess the sample integrity. The mRNA-seq library preparation from 1 μg of total RNA was performed with TruSeq RNA Sample Prep Kit (Illumina, USA) according to the manufacturer's instructions. The library fragments were purified with AMPure XP system (Beckman, USA) to select cDNA fragments of preferentially 250-300bp. All libraries were sequenced on the Illumina Hiseq 2500 (Novogene Biotech, China) for about 20-30 million paired-end reads with a fragment length of 150bp per sample. Raw data were deposited in the NCBI GEO repository under the accession number GSE158104 (https://www.ncbi.nlm.nih.gov/geo/query/acc.cgi?acc=GSE158104).

### 2.2 Chromatin immunoprecipitation library construction and sequencing

Chromatin was prepared from the newly regenerated heart tissues of zebrafish at 0 dpa, 6 dpa and 9 dpa following the manual instruction of ChIP-IT High Sensitive Kit (Active Motif, belgium). For each sample, frozen tissue was cut into small pieces on dry ice and transferred to 1.5 ml tubes containing 2% (w/v) formaldehyde in PBS, and then cross-linked at room temperature for 20 min. Thereafter, fixed tissues were crushed in cold lysis buffer [10 mM Tris-HCl (pH 7.4), 10 mM NaCl, 3 mM MgCl2 (Sangon Biotech, China), and 0.1% IGEPAL CA-630 (Sigma-Aldrich, USA)] on ice for 10-20 min to prepare nuclear extracts. Then sonication (Covaris S220, USA) was performed to shear the chromatin to 100-300 bp, and lysates were immunoprecipitated overnight at 4°C with protein A Dynabeads (Invitrogen, USA) coupled with 3-5 μg of antibody specific for H3K9ac or H3K9me3 (Abcam, USA). Recovered DNA was analyzed by quantitative PCR (ChIP-qPCR) and sequencing (ChIP-Seq). ChIP-DNA was purified using a QIAquick PCR Purification Kit (QIAGEN, Germany), ChIP-seq libraries were generated by ThruPLEX-FD Prep Kit (Rubicon, USA), and sequenced by Illumina Hiseq 2500 (Novogene Biotech, China) using the paired-end module and with 150 bp reads on each end. Raw data were deposited in the NCBI GEO repository under the accession number GSE158104 (https://www.ncbi.nlm.nih.gov/geo/query/acc.cgi?acc=GSE158104).

### 2.3 RNA-seq data analysis

Processing of RNA-seq data was carried out as described previously [7,8]. The Illumina FASTQ output files of sequences were assessed for quality control using FastQC (version 0.11.6). The sequences were then aligned to the zebrafish genome build danRer10 genome using STAR aligner (https://github.com/alexdobin/STAR) [1]. The mapped reads were converted to FPKM (fragments per kilobase million reads) by running Cufflink tools [2] to quantify mRNA expression levels. Differential expression genes (DEGs) were filtered for a P value < 0.05, the absolute value of fold change (FC) >= 1.5 and the average FPKM >= 3 in at least one of the two groups based on R package edgeR. SAMtools [3] was used to filter paired reads and remove duplicate reads from mapped BAM files and converted into BigWig format using “bamCoverage” script from deepTools 2.0 [6]. Descriptive statistics on the RNA-seq data of the 6 samples are provided in Table 1.

### 2.4 ChIP-seq data analysis

Raw ChIP-seq data were assessed for quality control using FastQC (version 0.11.6). The sequenced reads were then trimmed and aligned to zebrafish genome build danRer10 genome using Bowtie2 aligner [4] with parameters -X 1000 -5 1 -3 70. Duplicate reads were removed and BAM files were generated with SAM tools. The module “callpeak” in MACS2 (Model-based Analysis for ChIP Sequencing v2.0) [5] was used to identify regions of ChIP-seq enrichment over the background in an unbiased manner. For histone modification H3K9ac and H3K9me3 ChIP-seq data, the parameters were modified to facilitate accurate detection of broad peaks with –broad –broad-cutoff 1E-3 -p 1E-5. For normalization and visualization, the filtered, sorted BAM files were converted to BigWig format using the “bamCoverage” scripts in deepTools v2.0 [6] with parameters -bs 20 –normalizeUsing RPKM. Descriptive statistics on the ChIP-seq data of the 6 samples are given in Table 2 and Table 3.

## Ethics Statements

All zebrafish were reared and used for the experiments in accordance with the Animal Care and Use Committee guidelines of Shanghai Ocean University (SHOU-DW-2012-082).

## CRediT authorship contribution statement

**Xuelong Wang:** Conceptualization, Formal analysis, Methodology, Investigation, Visualization, Writing – review & editing. **Huiping Guo:** Formal analysis, Methodology, Investigation, Writing – review & editing. **Feifei Yu:** Formal analysis, Methodology, Investigation. **Hui Zhang:** Methodology, Investigation. **Ying Peng:** Methodology, Investigation. **Chenghui Wang:** Investigation, Funding acquisition, Writing – review & editing. **Gang Wei:** Investigation, Funding acquisition, Writing – review & editing. **Jizhou Yan:** Conceptualization, Formal analysis, Funding acquisition, Writing – review & editing.

## Declaration of Competing Interest

The authors declare that they have no known competing financial interests or personal relationships that could have appeared to influence the work reported in this paper.

## Data Availability

Dataset for transcriptomic, H3K9ac and H3K9me3 profiles during cardiac regeneration in zebrafish (Original data) (NCBI GEO repository).

## Appendix Supplementary materials

Image, application 1

## References

[1] Dobin A., Davis C.A., Schlesinger F., Drenkow J., Zaleski C., Jha S., Batut P., Chaisson M., Gingeras T.R. (2013). STAR: ultrafast universal RNA-seq aligner. Bioinformatics.

[2] Trapnell C., Roberts A., Goff L.A., Pertea G., Kim D., Kelley D.R., Pimentel H., Salzberg S.L., Rinn J.L., Pachter L. (2012). Differential gene and transcript expression analysis of RNA-seq experiments with TopHat and Cufflinks. Nat. Protoc..

[3] Li H., Handsaker B., Wysoker A., Fennell T., Ruan J., Homer N., Marth G., Abecasis G., Durbin R., Genome S. (2009). Project Data Processing, The Sequence Alignment/Map format and SAMtools. Bioinformatics.

[4] Langmead B., Salzberg S.L. (2012). Fast gapped-read alignment with Bowtie 2. Nat. Methods.

[5] Zhang Y., Liu T., Meyer C.A., Eeckhoute J., Johnson D.S., Bernstein B.E., Nusbaum C., Myers R.M., Brown M., Li W., Liu X.S. (2008). Model-based analysis of ChIP-Seq (MACS). Genome Biol..

[6] Ramirez F., Dundar F., Diehl S., Gruning B.A., Manke T. (2014). deepTools: a flexible platform for exploring deep-sequencing data. Nucleic Acids Res..

[7] Wang X., Yan J., Shen B., Wei G. (2021). Integrated Chromatin Accessibility and Transcriptome Landscapes of Doxorubicin-Resistant Breast Cancer Cells. Front. Cell Dev. Biol..

[8] Wang X., Guo H., Yu F., Zhang H., Peng Y., Wang C., Wei G., Yan J. (2022). Keratin5-cytoskeleton-BMP4 network regulates cell phenotype conversions during cardiac regeneration. Exp. Cell Res..

