# Supplementary material for: Dataset for transcriptomic, H3K9ac and H3K9me3 profiles during cardiac regeneration
Source: Data Brief. 2022 Sep 2;45:108569. doi: 10.1016/j.dib.2022.108569 (PMC9483719; doi:10.1016/j.dib.2022.108569)
Supplement: Supplementary file 1 [file mmc1.zip › mRNA_0dpa_rep1_R2_fastqc.html]

mRNA\_0dpa\_rep1\_R2.fastq.gz FastQC Report 

FastQC Report

周六 23 7月 2022  
mRNA\_0dpa\_rep1\_R2.fastq.gz

## Summary

- Basic Statistics
- Per base sequence quality
- Per tile sequence quality
- Per sequence quality scores
- Per base sequence content
- Per sequence GC content
- Per base N content
- Sequence Length Distribution
- Sequence Duplication Levels
- Overrepresented sequences
- Adapter Content

## Basic Statistics

| Measure | Value |
| --- | --- |
| Filename | mRNA\_0dpa\_rep1\_R2.fastq.gz |
| File type | Conventional base calls |
| Encoding | Sanger / Illumina 1.9 |
| Total Sequences | 26000581 |
| Sequences flagged as poor quality | 0 |
| Sequence length | 151 |
| %GC | 47 |

## Per base sequence quality

## Per tile sequence quality

## Per sequence quality scores

## Per base sequence content

## Per sequence GC content

## Per base N content

## Sequence Length Distribution

## Sequence Duplication Levels

## Overrepresented sequences

No overrepresented sequences

## Adapter Content

Produced by FastQC (version 0.11.8)
