# Supplementary material for: Dataset for transcriptomic, H3K9ac and H3K9me3 profiles during cardiac regeneration
Source: Data Brief. 2022 Sep 2;45:108569. doi: 10.1016/j.dib.2022.108569 (PMC9483719; doi:10.1016/j.dib.2022.108569)
Supplement: Supplementary file 1 [file mmc1.zip › H3K9me3_0dpa_R1_fastqc.html]

H3K9me3\_0dpa\_R1.fastq.gz FastQC Report 

FastQC Report

周六 23 7月 2022  
H3K9me3\_0dpa\_R1.fastq.gz

## Summary

- Basic Statistics
- Per base sequence quality
- Per tile sequence quality
- Per sequence quality scores
- Per base sequence content
- Per sequence GC content
- Per base N content
- Sequence Length Distribution
- Sequence Duplication Levels
- Overrepresented sequences
- Adapter Content

## Basic Statistics

| Measure | Value |
| --- | --- |
| Filename | H3K9me3\_0dpa\_R1.fastq.gz |
| File type | Conventional base calls |
| Encoding | Sanger / Illumina 1.9 |
| Total Sequences | 34407755 |
| Sequences flagged as poor quality | 0 |
| Sequence length | 151 |
| %GC | 45 |

## Per base sequence quality

## Per tile sequence quality

## Per sequence quality scores

## Per base sequence content

## Per sequence GC content

## Per base N content

## Sequence Length Distribution

## Sequence Duplication Levels

## Overrepresented sequences

| Sequence | Count | Percentage | Possible Source |
| --- | --- | --- | --- |
| AGATCGGAAGAGCACACGTCTGAACTCCAGTCACATCACGATCTCGTATG | 7453145 | 21.661235962648536 | TruSeq Adapter, Index 1 (100% over 49bp) |
| AGATCGGAGAGCACACGTCTGAACTCCAGTCACATCACGATCTCGTATGC | 138269 | 0.4018541750253685 | TruSeq Adapter, Index 1 (100% over 43bp) |
| AGATCGGAAGAGCACACGTCTGAACTCCAGTCACATCACGATCGCGTATG | 122805 | 0.35691081850588624 | TruSeq Adapter, Index 1 (97% over 49bp) |
| AGATCGAAGAGCACACGTCTGAACTCCAGTCACATCACGATCTCGTATGC | 87477 | 0.2542362906269241 | TruSeq Adapter, Index 1 (97% over 46bp) |
| AGATCGGAAGAGCACACGAGATCGGAAGAGCACACGTCTGAACTCCAGTC | 81638 | 0.23726627906993641 | Illumina Multiplexing PCR Primer 2.01 (100% over 32bp) |
| AGATCGGAAGAGCACACGTCTGAACTCCAGTCACATCACGATCACGTATG | 68246 | 0.19834482081147112 | TruSeq Adapter, Index 1 (97% over 49bp) |
| AGATCGGAAGAGCACACGTCTGAACTCCAGTCACATCACGATCCCGTATG | 61413 | 0.17848592562926585 | TruSeq Adapter, Index 1 (97% over 49bp) |
| GATCGGAAGAGCACACGTCTGAACTCCAGTCACATCACGATCTCGTATGC | 59837 | 0.1739055628593031 | TruSeq Adapter, Index 1 (100% over 50bp) |
| GAAGAGCACACGTCTGAACTCCAGTCACATCACGATCTCGTATGCCGTCT | 46239 | 0.13438540236060156 | TruSeq Adapter, Index 1 (100% over 50bp) |
| CCGATCTAGATCGGAAGAGCACACGTCTGAACTCCAGTCACATCACGATC | 43078 | 0.12519851992668513 | TruSeq Adapter, Index 1 (100% over 42bp) |

## Adapter Content

Produced by FastQC (version 0.11.8)
