# Supplementary material for: Dataset for transcriptomic, H3K9ac and H3K9me3 profiles during cardiac regeneration
Source: Data Brief. 2022 Sep 2;45:108569. doi: 10.1016/j.dib.2022.108569 (PMC9483719; doi:10.1016/j.dib.2022.108569)
Supplement: Supplementary file 1 [file mmc1.zip › H3K9me3_0dpa_R2_fastqc.html]

H3K9me3\_0dpa\_R2.fastq.gz FastQC Report 

FastQC Report

周六 23 7月 2022  
H3K9me3\_0dpa\_R2.fastq.gz

## Summary

- Basic Statistics
- Per base sequence quality
- Per tile sequence quality
- Per sequence quality scores
- Per base sequence content
- Per sequence GC content
- Per base N content
- Sequence Length Distribution
- Sequence Duplication Levels
- Overrepresented sequences
- Adapter Content

## Basic Statistics

| Measure | Value |
| --- | --- |
| Filename | H3K9me3\_0dpa\_R2.fastq.gz |
| File type | Conventional base calls |
| Encoding | Sanger / Illumina 1.9 |
| Total Sequences | 34407755 |
| Sequences flagged as poor quality | 0 |
| Sequence length | 151 |
| %GC | 45 |

## Per base sequence quality

## Per tile sequence quality

## Per sequence quality scores

## Per base sequence content

## Per sequence GC content

## Per base N content

## Sequence Length Distribution

## Sequence Duplication Levels

## Overrepresented sequences

| Sequence | Count | Percentage | Possible Source |
| --- | --- | --- | --- |
| AGATCGGAAGAGCGTCGTGTAGGGAAAGAGTGTAGATCTCGGTGGTCGCC | 7663054 | 22.271299013841503 | Illumina Single End PCR Primer 1 (100% over 50bp) |
| CGTGTGCTCTTCCGATCTAGATCGGAAGAGCGTCGTGTAGGGAAAGAGTG | 88383 | 0.25686941795534174 | Illumina Single End PCR Primer 1 (100% over 32bp) |
| GATCGGAAGAGCGTCGTGTAGGGAAAGAGTGTAGATCTCGGTGGTCGCCG | 60922 | 0.1770589217459843 | Illumina Single End PCR Primer 1 (100% over 50bp) |
| GAAGAGCGTCGTGTAGGGAAAGAGTGTAGATCTCGGTGGTCGCCGTATCA | 42945 | 0.12481197915993066 | Illumina Single End PCR Primer 1 (100% over 50bp) |
| AGATCGGAGATCGGAAGAGCGTCGTGTAGGGAAAGAGTGTAGATCTCGGT | 42141 | 0.12247529663007657 | Illumina Single End PCR Primer 1 (100% over 43bp) |

## Adapter Content

Produced by FastQC (version 0.11.8)
